# Predicting student outcomes using digital logs of learning behaviors: Review, current standards, and suggestions for future work

**DOI:** 10.3758/s13428-022-01939-9

**Published:** 2022-08-26

**Authors:** Cara J. Arizmendi, Matthew L. Bernacki, Mladen Raković, Robert D. Plumley, Christopher J. Urban, A. T. Panter, Jeffrey A. Greene, Kathleen M. Gates

**Affiliations:** 1 Duke University, Durham, NC USA; 2 The University of North Carolina Chapel Hill, Chapel Hill, NC USA; 3 Centre for Learning Analytics, Monash University, Melbourne, Australia

**Keywords:** Digital data, Learning management system, Machine learning, Equity, Data privacy

## Abstract

Using traces of behaviors to predict outcomes is useful in varied contexts ranging from buyer behaviors to behaviors collected from smart-home devices. Increasingly, higher education systems have been using Learning Management System (LMS) digital data to capture and understand students’ learning and well-being. Researchers in the social sciences are increasingly interested in the potential of using digital log data to predict outcomes and design interventions. Using LMS data for predicting the likelihood of students’ success in for-credit college courses provides a useful example of how social scientists can use these techniques on a variety of data types. Here, we provide a primer on how LMS data can be feature-mapped and analyzed to accomplish these goals. We begin with a literature review summarizing current approaches to analyzing LMS data, then discuss ethical issues of privacy when using demographic data and equitable model building. In the second part of the paper, we provide an overview of popular machine learning algorithms and review analytic considerations such as feature generation, assessment of model performance, and sampling techniques. Finally, we conclude with an empirical example demonstrating the ability of LMS data to predict student success, summarizing important features and assessing model performance across different model specifications.

There has been growing interest in the timely identification of students who are likely to perform poorly in for-credit Science, Technology, Engineering, and Math (STEM) classes. Once identified, interventions attempting to prevent attrition can be made (Pistilli, Willis, & Campbell, 2014; Pritchard & Wilson, 2003; Zajacova, Lynch, & Espenshade, 2005; Cogliano, Bernacki, Hilpert, & Strong, 2022). Identifying students is particularly important in STEM disciplines given the high attrition rates of students typically underrepresented in these fields (National Academies of Sciences Engineering & Medicine, 2016) and the threats to the supply of qualified STEM professionals that attrition brings (Dai & Cromley, 2014). Several variables derived from different data sources have previously been used to predict students’ likelihood of success and failure. Those variables often include demographic information about underrepresented groups (e.g., Dennis, Phinney, & Chuateco, 2005; Petty, 2014; Pritchard & Wilson, 2003; Tinto, 1975; Tinto & et al. 1993), which is currently under an ethical debate (Tene & Polonetsky, 2013; Slade & Tait, 2019), and self-report data (e.g., about motivation; Hulleman, Godes, Hendricks, & Harackiewicz, 2010). Increasingly, researchers have utilized technology-enhanced learning environments (e.g., learning management systems; LMS) to collect data about real-time learning behaviors that relate to distal course performance (Macfadyen & Dawson, 2010; Márquez-Vera, Cano, Romero, & Ventura, 2013). The prediction of for-credit course performance based on LMS data provides a useful example for social scientists interested in digital trace data as a source of behavioral data. This paper adds to research on predictive modeling for student success using LMS data while also demonstrating how digital trace data may be used by researchers in general.

LMS has increasingly become a commonplace tool in higher education (Malloy, Jensen, Regan, & Reddick, 2002). Instructors use the platform for communicating with students, conducting critical assessment tasks, and sharing digital resources students can use for learning. When instructors and students make use of LMS features, the system captures a trace of each event in a log file. The trace data can potentially allow researchers to better understand learning behaviors of students as they provide a rich, fine-grained, and accurate record of students’ actions (Nistor & Neubauer, 2010). These passively collected data provide potential utility for a wide range of higher education institutes hoping to decrease attrition rates in specific fields.

The log data collected through an LMS provide some advantages in predicting student achievement. LMSs such as Blackboard Learn, Canvas, Desire2Learn, or Sakai capture and store learning activities of students with time stamps at a fine-grained level, allowing researchers to track a variety of user actions and to examine the data from various perspectives (Krumm, Waddington, Teasley, & Lonn, 2014). Namely, the data contains a large amount of information including frequency, time, and patterns of a series of activities (e.g., reading, posting, and taking exams) that reflect learning processes (Black, Dawson, & Priem, 2008; Bernacki, 2018). Importantly, the data are obtained in a naturalistic setting, allowing researchers insight into real-life learning behaviors. Much of the interesting information in this kind of research, based on this emerging data type, could not be obtained by other means.

With the increased availability of such data comes the increase in its use by researchers and educators. Those new to this kind of approach will find themselves facing a number of questions and a wide variety of decision-points specific to the data type. The present paper serves as a resource to aid in understanding the opportunities and hurdles present when using LMS data to predict student success in a specific course, but ideas in this paper can be expanded to other types of digital trace data. We begin by providing an extensive overview of the use of LMS data to date. In this review, we outline the types of methods and variables used, as well as highlight some of the major outcomes. Using these prior studies as a foundation, we next focus on suggestions for researchers as the field moves forward. We discuss ethical considerations in the use of these data that provide a backdrop for each decision point to come. We also provide an overview of a few approaches for analyzing the data. As those methods are continuously being improved upon, we focus less on the specific approaches and more on qualities of data mining approaches that researchers should consider when selecting an algorithm. Finally, and perhaps most critically, we discuss the critical role of feature construction and the options available in digital trace data. We use an exemplar dataset obtained on a student sample enrolled in an entry level undergraduate biology course to illustrate these points.

## Systematic literature review

To help inform the generation of useful features from digital trace behaviors and to gain insight into the expected performance rates for varied analytic approaches, we conducted an extensive literature review of publications that had similar goals to our project. Specifically, we searched for publications reporting on predictive models developed to predict students’ success in a single course. For reviews on other topics important to predictive modeling in education, we recommend Baker and Hawn (2021) for their review of algorithmic bias in education, (Paquette, Li, Baker, Ocumpaugh, & Andres, 2020) for their review of the use of demographics variables in educational data mining, and Kizilcec and Lee (2021) for their review of algorithmic fairness in education.

We queried EBSCO and Google Scholar bibliographical databases with the search phrase ”predict student success AND higher education”. The term ”course success” was used as an additional phrase in searching Google Scholar to narrow down the number of results returned. After removing duplicates of the returned results, the abstracts of 381 publications were screened for eligibility and 75 publications were selected for detailed assessment. As per our inclusion criteria, we selected only those studies that (1) focused on undergraduate students, rather than on other populations of learners, and (2) investigated models that predicted students’ course-specific performance, rather than other variables often used as an indicator of ”success” (e.g., course satisfaction or engagement, or broader academic performances that span multiple classes). We limited the included studies to those that predicted course-specific success because the focus of this review was prediction modeling using behavioral digital trace data. Behavioral digital trace data are very course-specific (e.g., downloads of a particular course resource or accesses of a course-specific tool) and/or situated (e.g., timing of accessing a course gradebook after an exam), and as such there are few behavioral digital trace variables that would be common or similarly predictive across courses. Studies that investigate predictors of success that span multiple classes must necessarily use predictor variables that are broader than the digital trace data upon which our paper is focused (e.g., demographics) and/or indicators of success that are broader than our focus on specific course grades (e.g., grade point average, retention status). Therefore, such studies are not informative for our review and were excluded. After close reading of 75 publications, 39 met these criteria and received full review. The other 36 publications were excluded due to a lack of clarity in operationalizing or measuring student success or a failure to report any kind of performance metrics for the predictive models. This review is up to date as of October 2020.

Across the 39 papers, we identified 82 different predictive models and report them in Table 1. Models relied on a variety of demographic, performance, and behavioral variables to predict the probability of students’ academic success in a course. Most of the models were designed to predict a dichotomous outcome associated with a performance above or below a course-specific cutpoint of meaning to those enrolled (e.g., a B or Better vs. a C or Worse, at/above vs. below class median, safe vs. at-risk, pass vs. fail, or successful vs. not successful; Baker, Lindrum, Lindrum, & Perkowski, 2015; Wolff, Zdrahal, Nikolov, & Pantucek, 2013; Zacharis, 2015; Bernacki, Chavez, & Uesbeck, 2020; Yu, Li, Fischer, Doroudi, & Xu, 2020). Reports of model performance most often relied on prediction accuracy, which provides a rate of true positives and negatives as one value, as a sole performance measure. Additional reported metrics included specificity (i.e., model’s ability to detect true negative outcomes) and precision (i.e., model’s ability to detect true-positive outcomes). These metrics provide additional insight into the kinds of classification accuracy achieved by models and appear in the table. Other papers also reported the following metrics: F-score (Lee & Kizilcec, 2020; Wolff et al., 2013) and false-positive/false-negative rate (Yu et al., 2020). We chose to focus on accuracy because it was the most commonly reported metric among all prediction models found in our search.

**Table 1:** Data sources and the accuracy achieved in studies reporting prediction of higher education outcomes (mean accuracy = 71.6%)

			Predictors		Outcome					
Authors	Data sources	Demographic	Static/baseline	Behavioral	Variable	Level	Algorithm	Performance measures (accuracy / precision / specificity)	Cross-validation	Observed (Weeks)
Baker et al., (2015)	Soomo learning environment	-	-	X	pass or fail	category (binary)	Logistic regression	.66/.57/.60	cross-validation	4
Baker et al., (2015)	Soomo learning environment	-	-	X	pass or fail	category (binary)	Decision tree (J-48)	.66/.64/.44	cross-validation	4
Baker et al., (2015)	Soomo learning environment	-	-	X	pass or fail	category (binary)	Decision tree (J-Rip)	.58/.57/.47	cross-validation	4
Baker et al., (2015)	Soomo learning environment	-	-	X	pass or fail	category (binary)	Naïve Bayes	.67/.53/.48	cross-validation	4
Baker et al., (2015)	Soomo learning environment	-	-	X	pass or fail	category (binary)	W-K-Star	.68/.67/.28	cross-validation	4
Baker et al., (2015)	Soomo learning environment	-	-	X	pass or fail	category (binary)	Stepwise multiple regression	.66/.57/.60	cross-validation	4
Barber & Sharkey (2012)	LMS, financial aid system, student system	gender, age, military status, financial aid receipt, ethnicity	X	X	risk of failing	category (high, low, neutral)	Logistic regression	>.90/-/-	50-50 train/test	4
Barber & Sharkey (2012)	LMS, financial aid system, student system	gender, age, military status, financial aid receipt, ethnicity	X	X	risk of failing	category (high, low, or neutral)	Naïve Bayes	.85-.95/-/-	Number of folds not provided, repeated ten times	4
Bernacki et al., (2020)	LMS (M1)	-	-	X	pass or fail	category (binary)	Logistic regression	.61/.41./76	leave-one-out and 10-fold	4
Bernacki et al., (2020)	LMS (M1)	-	-	X	pass or fail	category (binary)	Decision tree (J-48)	.54/.44/.62	leave-one-out and 10-fold	4
Bernacki et al., (2020)	LMS (M1)	-	-	X	pass or fail	category (binary)	Decision tree (J-Rip)	.64/.48/.77	leave-one-out and 10-fold	4
Bernacki et al., (2020)	LMS (M1)	-	-	X	pass or fail	category (binary)	Naïve Bayes	.61/.35/.80	leave-one-out and 10-fold	4
Bernacki et al., (2020)	LMS (M2)	-	-	X	pass or fail	category (binary)	Logistic regression	.67/.55/.77	leave-one-out and 10-fold	4
Bernacki et al., (2020)	LMS (M2)	-	-	X	pass or fail	category (binary)	Decision tree (J-48)	.59/.40/.74	leave-one-out and 10-fold	4
Bernacki et al., (2020)	LMS (M2)	-	-	X	pass or fail	category (binary)	Decision tree (J-Rip)	.65/.50/.77	leave-one-out and 10-fold	4
Bernacki et al., (2020)	LMS (M2)	-	-	X	pass or fail	category (binary)	Naïve Bayes	.66/.59/.71	leave-one-out and 10-fold	4
Bernacki et al., (2020)	LMS (M3)	-	-	X	pass or fail	category (binary)	Logistic regression	.67/.57/.75	leave-one-out and 10-fold	4
Bernacki et al., (2020)	LMS (M3)	-	-	X	pass or fail	category (binary)	Decision tree (J-48)	.61/.37/.80	leave-one-out and 10-fold	4
Bernacki et al., (2020)	LMS (M3)	-	-	X	pass or fail	category (binary)	Decision tree (J-Rip)	.63/.48/.75	leave-one-out and 10-fold	4
Bernacki et al., (2020)	LMS (M3)	-	-	X	pass or fail	category (binary)	Naïve Bayes	.65/.53/.74	leave-one-out and 10-fold	4
Bird (2012)	-	ethnicity, age, class	X	-	successful vs. not successful	category (binary)	Logistic regression	-/0/100	-	-
Bird (2012)	-	ethnicity, age, class, distance from the university	X	-	successful vs. not successful	category (binary)	Discriminant analysis	-/.50/.90	-	-
Cakmak (2017)	university database	-	X	-	course grade	category	Collaborative filtering	.74/-/-	details not provided	-
Choi et al., (2018)	classroom response system, survey instrument	-	X	X	level of exam score	category	Hierarchical linear regression (R2 = 0.57)	.72/.60/.47	10-fold, repeated 20 times	1
Choi et al., (2018)	classroom response system, survey instrument	-	X	X	level of exam score	category	Hierarchical linear regression (R2 = 0.57)	.73/.60/.47	10-fold, repeated 20 times	3
Choi et al., (2018)	classroom response system, survey instrument	-	X	X	level of exam score	category	Hierarchical linear regression (R2 = 0.57)	.80/.70/.58	10-fold, repeated 20 times	6
Choi et al., (2018)	classroom response system, survey instrument	-	X	X	level of exam score	category	Hierarchical linear regression (R2 = 0.57)	.82/.75/.61	10-fold, repeated 20 times	9
Choi et al., (2018)	classroom response system, survey instrument	-	X	X	level of exam score	category	Hierarchical linear regression (R2 = 0.57)	.82/.75/.61	10-fold, repeated 20 times	12
Choi et al., (2018)	classroom response system, survey instrument	-	X	X	pass or fail	category (binary)	Hierarchical logistic regression (RMSE= 0.89)	.72/.44/.45	10-fold, repeated 20 times	1
Choi et al., (2018)	classroom response system, survey instrument	-	X	X	pass or fail	category (binary)	Hierarchical logistic regression (RMSE= 0.89)	.73/.49/.47	10-fold, repeated 20 times	3
Choi et al., (2018)	classroom response system, survey instrument	-	X	X	pass or fail	category (binary)	Hierarchical logistic regression (RMSE= 0.89)	.80/.58/.60	10-fold, repeated 20 times	6
Choi et al., (2018)	classroom response system, survey instrument	-	X	X	pass or fail	category (binary)	Hierarchical logistic regression (RMSE= 0.89)	.82/.63/.66	10-fold, repeated 20 times	9
Choi et al., (2018)	classroom response system, survey instrument	-	X	X	pass or fail	category (binary)	Hierarchical logistic regression (RMSE= 0.89)	.82/.63/.66	10-fold, repeated 20 times	12
Cooper & Pearson (2012)	student information database	race	X	-	safe or at-risk	category (binary)	Neural network	.83/.76/-	genetic optimization, leave-one-out cross validation	-
Culver (2014)	student information system, math assessment and college student inventory form	gender, race, diploma type, years since high school	X	-	final grade	category	Logistic regression	.62/-/-	not provided	-
Cummings (2009)	university database	age, gender, number of semesters at college	X	-	course grade	successful vs. not successful	Logistic regression	.72/.89/.96	not provided	-
D’Aloisio (2016)	survey instruments	gender, high school GPA, number of hours per week spent on mathematics	X	-	final average score	continuous	Multiple regression (R2 = 0.44)	-/-/-	not provided	15
Das (2009)	survey	-	-	-	midterm grade	category	Multiple regression (R2 = 0.17)	-/-/-	not provided	6
Das (2009)	survey	-	X	-	midterm grade	category	Logistic regression	.87/-/-	not provided	6
Davidson (2017)	student information system	age, gender, ethnicity	X	-	successful vs. not successful	category (binary)	Logistic regression	.65/-/-	not provided	-
Davidson (2017)	student information system	age, gender, ethnicity	X	-	successful vs. not successful	category (binary)	Logistic regression	.81/-/-	not provided	-
Davidson (2017)	student information system	age, gender, ethnicity	X	-	successful vs. not successful	category (binary)	Logistic regression	.85/-/-	not provided	-
Davidson (2017)	student information system	age, gender, ethnicity	X	-	successful vs. not successful	category (binary)	Logistic regression	.80/-/-	not provided	-
Fogle (2016)	archival records	age, gender, and ethnicity	X	-	final grade	category (binary)	Logistic regression (R2 = 0.30)	-/-/-	not provided	-
Fountain (2016)	survey instruments, class records	gender, race/ethnicity, age, enrollment status, student and online experience	-	-	c or higher vs. lower	category (binary)	Logistic regression	.85/-/-	not provided	-
Goad (2018)	survey instruments	-	X	-	completing or not	category (binary)	Logistic regression	.73/-/-	not provided	2
Goosen (2008)	-	age, gender, and ethnicity	X	-	exit examination score	continuous	Stepwise multiple regression (R2 = 0.13)	-/-/-	not provided	-
Gorvine & Smith (2015)	survey instruments	attitudes towards group work and statistics	-	-	percentage points	continuous	Hierarchical regression analysis (R2 = 0.06)	-/-/-	not provided	-
Gultice et al., (2015)	survey instruments	age, hours earned/carried	X	-	pass or fail	category (binary)	Logistic regression	.82/.64/.91	10-fold stratified, ten repetitions	1
Hauser, (2016)	academic services	age, gender, ethnicity, prior online course experience, and enrollment status during the semester	-	-	final grade	category (binary)	Logistic regression	.83/-/-	not provided	-
Junco & Clem (2015)	CourseSmart eTextbook, university records	gender, race, ethnicity	X	X	final score	continuous	Blocked linear regression (R2 = 0.24)	-/-/-	Not provided	16
Kotsiantis et al., (2013)	LMS logs registrar	-	-	X	pass or fail	category (binary)	C4.5 (decision tree)	. 82/-/-	10-fold, repetition not provided	11
Lee (2016)	ToOLS assessment	-	X	-	successful vs. not successful	category (binary)	Nested regression (R2 = 0.45)	-/-/-	not provided	-
Lee & Kizilcec (2020)	student administrative data	Gender, first-generation status, racial-ethnic group	X	-	a course grade at/ above course median grade vs. not	category (binary)	Random Forest	0.73/-/- (F-score=.80)	-	-
McFate & Olmsted III (1999)	not reported	-	X	-	completing or not	category (binary)	Correlational analysis (R2 = 0.82)	-/-/-	Not provided	1
Morrison & Schmit (2010)	-		X	-	successful vs. not successful	category (binary)	Logistic regression (R2 = 0.31)	-/-/-	Not provided	-
O’Connell et al., (2018)	-	pell eligible, race, completed hours	X	X	course grade	discrete grades (a-f)	Multiple regression	.58/-/-	best-subsets model selection	-
O’Connell et al., (2018)	-	Pell eligible, race, completed hours	X	X	pass or fail	category (binary)	Logistic regression	.47/-/-	best-subsets model selection	-
Ornelas & Ordonez (2017)	LMS, college database	-	X	X	successful vs. at-risk	category (binary)	Naïve Bayes	.94/.92/.95	training and validation set (60 and 40 %, respectively)	16
Rayno (2010)	archival records	age, gender, and ethnicity	X	-	pass or fail	category (binary)	Logistic regression	.81/-/-	not provided	-
Romero et al., (2010)	LMS logs and database	-	-	X	final grade	category	Decision Tree (CART)	.66/-/-	10-fold stratified, ten repetitions	-
Romero et al., (2010)	LMS logs and database	-	-	X	final grade	category	Decision Tree (C.45)	.65/-/-	10-fold stratified, ten repetitions	-
Romero et al., (2010)	LMS logs and database	-	-	X	final grade	category	Rule Induction	.65/-/-	10-fold stratified, ten repetitions	-
Saqr et al., (2017)	LMS	-	X	X	final grade	continuous (0 to 100)	Automatic linear modeling (ALM)	.64/.54/-	Not provided	6
Saqr et al., (2017)	LMS	-	X	X	safe or at-risk	category (binary)	Binary logistic regression (R2 = 0.77)	.81/-/-	Not provided	6
Smith et al., (2012)	LMS logs, registrar	-	X	X	risk of failing	category (warning levels: low, moderate, high)	Naïve Bayes	70 in Low, 54 in Moderate, 34 in the High warning group/-/-	Random sub-sampling, repeated ten times	-
Williams (2019)	VitalSource	-	-	X	final grade	category	Multiple regression (R2 = 0.15)	-/-/-	not provided	8
Williams (2019)	VitalSource	-	-	X	average test score	continuous	Multiple regression (R2 = 0.10)	-/-/-	not provided	8
Wolff et al., (2013)	virtual learning environment, registrar	used in the model, but not reported	X	X	pass or fail	category (binary)	Decision trees	F=[0.61, 0.94], tested at the three different assessment submission points	10-fold, repetitions data not provided	1 period between Tutor marked Assessments
Xing, Guo, Petakovic, & Goggins (2015)	CSCL environment; event logs	-	-	X	course performance	category (five-point scale)	Genetic Programming	.80/.80/-	10-fold, repeated ten times	-
You (2016)	LMS	-	-	X	final score	continuous (0 to 100)	Hierarchical regression analysis (R2=.32)	.69/-/-	Not provided	8
Yu et al., (2020)	institutional data	gender, and transfer, income, first generation and URM status	X		short-term success (final course grade above the class median vs. not)	category (binary)	-	.62/-/- (FPR=.47, FNR=.30)	course-level leave- one-group-out cross validation	8
Yu et al., (2020)	Canvas LMS log data			X	short-term success (final course grade above the class median vs. not)	category (binary)	-	.60/-/- (FPR=.48, FNR=.31)	course-level leave- one-group-out cross validation	8
Yu et al., (2020)	survey data		X		short-term success (final course grade above the class median vs. not)	category (binary)	-	.53/-/-/(FPR=.60, FNR=.34)	course-level leave- one-group-out cross validation	8
Yu et al., (2020)	institutional data and Canvas LMS log data	gender, and transfer, income, first generation and URM status	X	X	short-term success (final course grade above the class median vs. not)	category (binary)	-	.67/-/-(FPR=.35, FNR=.31)	course-level leave- one-group-out cross validation	8
Yu et al., (2020)	institutional data and survey data	gender, and transfer, income, first generation and URM status	X	-	short-term success (final course grade above the class median vs. not)	category (binary)	-	.63/-/-(FPR=.40, FNR=.34)	course-level leave- one-group-out cross validation	8
Yu et al., (2020)	Canvas LMS log data and survey data		X	X	short-term success (final course grade above the class median vs. not)	category (binary)	-	.61/-/-(FPR=.43, FNR=.35)	course-level leave- one-group-out cross validation	8
Yu et al., (2020)	institutional data, Canvas LMS log data, and survey data	gender, and transfer, income, first generation and URM status	X	X	short-term success (final course grade above the class median vs. not)	category (binary)	-	.67/-/-(FPR=.35, FNR=.30)	course-level leave- one-group-out cross validation	8
Zabriskie et al., (2019)	institutional records and classes	gender, in-state, URM, First Generation	X	-	b or less/more	category (binary)	Random Forest	68/-/-	training and test set (62 and 38 %, respectively)	1
Zabriskie et al., (2019)	institutional records and classes	gender, in-state, URM, First Generation	X	-	b or less/more	category (binary)	Logistic regression	.73, .81 (two courses)/-/-	training and test set (62 and 38 %, respectively)	1
Zacharis (2015)	LMS	-	-	X	failed/did not fail	category (binary)	Logistic regression	.81/.70/.87	10-fold, repeated ten times	-
Zacharis (2018)	LMS logs, registrar	-	-	X	pass or fail	category (binary)	Decision tree	.99/.98/1	training and test set (70-30%)	-

### Current state and next steps

Across the models included in the table, the prediction accuracy spanned between .47 to .99, and the average was .72 (SD = .10). The number of weeks of a semester required to obtain this level of accuracy was 5.85 on average (SD = 3.67) and ranged from as few as 1 week to as many as 16. Our review highlighted three areas of improvement and areas of consideration for future studies, which we address in this paper. 1. Nearly two-thirds of the models that performed well included some demographic or static data rather than rely on the behavioral data. This raises some ethical concerns, to be discussed later. 2. Many studies utilized *only one* outcome measure (accuracy). 3. Newer analytic techniques, such as regularization, which carry benefits over traditional regression have not been used in these studies. One additional area of focus for future studies is how to best engineer the trace data obtained so that the features reach their maximum potential in terms of usefulness and power to predict, as well as the ways they might inform the kinds of interventions that would be apt to deploy as support for students’ learning. Next, we focus on these three critical areas in turn.

### Predictor variables used

#### Static variables

Among the types of predictors included in models, demographic data about students’ gender, race, and ethnicity were the most common. These data tend to be readily available to researchers as they are often solicited from learners during the enrollment process in higher education settings. However, the strength and direction of associations between demographic variables and course achievement are mixed across models. For example, gender’s role varies greatly across models where in one introductory biology course women are predicted to perform better (Hauser, 2016), in a developmental mathematics course they perform worse (Goosen, 2008), and in an introductory Algebra course, gender does not contribute to a prediction model (O’Connell, Wostl, Crosslin, Berry, & Grover, 2018). Similarly, students’ ethnic/racial minority status predicted success in one introductory biology course (Hauser, 2016), whereas ethnic/racial background was not predictive of course grade in an introductory programming course (Zacharis, 2015). First-generation college status was the least frequently studied demographic predictor. In the one study we found that included first-generation college status, this variable was minimally useful in predicting students as at-risk in a physics class (Zabriskie, Yang, DeVore, & Stewart, 2019).

Performance data were also used as predictors in many models. Common kinds of performance data found in our review were assessment scores (e.g., Choi, Lam, Li, & Wong, 2018; Cooper & Pearson, 2012; Saqr, Fors, & Tedre, 2017) and number of courses/hours completed and grades or scores earned (e.g., Barber & Sharkey, 2012; Culver, 2014; Bird, 2012). Some models included scores on placement exams as predictors (D’Aloisio, 2016; Culver, 2014; Gultice, Witham, & Kallmeyer, 2015; McFate & Olmsted, 1999).

Variables that describe students’ prior achievement can be useful contributors to models in that they capture students’ overall level of preparedness for a course, both in terms of course-specific prior knowledge (i.e., when the score reflects performance in a related, prerequisite course) or their general ability to successfully engage in similar learning tasks (e.g., undergraduate GPA to date). Performance data that reflect scores on early assignments within courses can be powerful predictors (e.g., Zabriskie et al., 2019), but care should be taken to not include variables as predictors if they also contribute mathematically to the criterion variable. In instances where models predict a course grade, the inclusion of scores on early assignments are confounded, rather than orthogonal and predictive (e.g., Saqr et al., 2017).

#### Behavioral (time-varying) variables

Behavioral trace data can be collected from usage logs from digital course resources (i.e., usually LMS and textbook companion sites) and provide a record of learning events that are theorized to predict, but are also orthogonal to, summative performance data (Bernacki et al., 2020). Examples of these behaviors are views, downloads, assignment submissions and forum contributions (e.g., Kotsiantis, Tselios, Filippidi, & Komis, 2013; Smith, Lange, & Huston, 2012; Zacharis, 2015; Bernacki et al., 2020), clicker responses in the classroom response system (Choi et al., 2018), and number of clicks in a virtual learning environment (Wolff et al., 2013).

Of the 82 predictive models reviewed in Table 1, 29 models made use of only behavioral predictors. For usage logs to be of use in predicting student success, theoretically aligned features need to be generated. In other words, in its raw form, user log data *are* messy and may not immediately confer meaningful measurement of behaviors. A typical user log consists of a timestamp along with an action taken (e.g., clicks, inputs of values into fields, page visits, and selections of dropdown menus). These user logs must be converted into a data type that can be modeled and makes theoretical sense. In one study where students were completing online courses through a learning environment, researchers explored the timeframe of learning environment access by creating a feature that indicated whether a student accessed the environment in the first *n* days of the course and how many days, *d*, it had been since last access on day *n* (Baker et al., 2015). This study also made use of performance on course exercises. Another study explored in-class features by aggregating weekly attendance to calculate the cumulative attendance rate along with cumulative in-class test scores (Choi et al., 2018). Other models also used weekly aggregation to arrive at features. For example, to create a feature, the number of discussion posts was aggregated weekly and then averaged for each individual to create a feature. Another example is getting a total count of messages that a student sent to the instructor (Barber & Sharkey, 2012).

Ultimately, methods for mapping captured events to a feature space should depend on the goals of a project. Important to the process of feature mapping is the use of metadata. Metadata helps researchers to be more specific about the behaviors under observation. For example, knowing that a student completed a download at three different timestamps may hold little predictive value. However, a download can be elaborated with metadata about the alignment of the timestamp to the semester and the annotation of the objects accessed to provide a human readable name that affords interpretation. One might thus be able to make more meaning from an event reflecting the download of a syllabus in the first week of the semester, as compared to observing a ”file download” event by a student on a specific date that is not understood as relevant to the academic context. In particular, we suggest researchers consider creating features from metadata that considers course content, behavioral specificity, and timeframe in which an action was completed. For example, if researchers or educators believe actions in particular course chapters or units hold differing predictive value, researchers may want to create features that break down page visits by course unit. Furthermore, the type of page visited in a given unit might hold importance. For example, a page visit to a practice test may be more predictive than a page visit to an additional reading. Further, researchers can isolate page visits to a particular time frame, such as 2 weeks before an exam. This feature can then defined be as number of downloads of a practice test 2 weeks prior to the exam. Another example is to dichotomize the feature to whether or not a student visited the practice test page 2 weeks prior to the exam. We provide more detail of feature examples with varying specificity, granularity, and timeframes in our presentation of the empirical example.

## Ethics in the use of LMS data for model building

One problem with approaching analysis with data-driven methods is that data science perspectives are often believed to be purely neutral and objective. This belief hides the reality that educational data mining (EDM) carries with it opportunity for threats to privacy as well as discrimination. It also involves a set of decisions that need to be documented and expressed to other researchers. The development of data-driven models to predict student success creates the potential for a number of well-intentioned but poorly reasoned decisions that can negatively impact the very students such models are meant to benefit. Negative impacts can arise from decisions regarding the privacy of student data, the collection of representative data sets from which models could be developed, the access to data describing learners and their actions, and the implementation of models that may perform inequitably for student subpopulations. New documents are now emerging in an effort to provide guidance to analysts who wish to develop models that can predict student success (e.g., Seldon, Lucking, Lakhani, & Clement-Jones, 2020), and we highlight two key elements that warrant consideration: privacy and use of demographic data.

### Privacy

Trace internet behaviors are used by many industries towards goals such as: predicting likelihood of purchase, modeling political opinion-formation processes, following the spread of emotion ”contagions” using social media data (Kaschesky & Riedl, 2011; Kramer, Guillory, & Hancock, 2014), and describing information flow using internet chain-letters (Liben-Nowell & Kleinberg, 2008). In these cases, there may be little to no expectation of privacy. For instance, when a user is navigating a store’s website, it is understood that the owner of that website will have access to their behaviors. The consent to use their data is implied by simply using the website, and due in no small part to that implied consent, internet behavioral tracking has emerged as a major asset in modern capitalism (Zuboff, 2015). Individuals have become comfortable trading aspects of their internet privacy for the benefits and conveniences brought by these services (Silverman, 2017). Such trade-offs are more complicated in higher education, and because of that, the writers of the Institute for Ethical AI in Education Interim Report (Seldon et al., 2020) argued data mining should not only follow a legal framework but also provide a clear benefit to students, have designated spaces without surveillance, and ensure that the collection of any health data have justification for how it helps educational purposes.

Despite the regularity and acceptance of gathering digital trace behaviors in other real-world contexts, the mere acquisition of such data can be intrusive and unexpected in the educational setting. The merging of data sets across multiple institutions has caused concern (Singer, 2014), as has the use of data even within one institute but across different data systems (Parry, 2012). Thus, whereas students may have accepted foregoing privacy when utilizing the internet for activities such as social media interactions and online shopping, the expectation for privacy may be greater when engaging in educational activities using LMS, particularly given students often have little recourse or knowledge in regard to their personal data management (Jerome, 2013; Tene & Polonetsky, 2013; Rubel & Jones, 2016). Many students rarely know what data are actually gathered about them and to what ends it may be used (Richards & King, 2013).

Many themes that we considered in this discussion are focal in the prediction modeling literature we reviewed, yet the authors of those papers did not focus on privacy as an issue. This may be due, in part, from prior research where researchers primarily studied deidentified data and archival data. In more recent years, a focus on data ownership and the importance of respecting student privacy has become a focal issue in learning analytics (Prinsloo & Slade, 2017; Zeide, 2017) especially in circumstances where data are collected in real time from multiple data systems and where ownership of and access to such data fall under the governance of university policies informed by local and national laws.

### Demographics

Student demographics relate to various outcomes of success in STEM disciplines. In STEM disciplines, fewer than 25% of underrepresented students, such as Latinx/Hispanic and African-American students, complete their stated major within 6 years (Eagan, Hurtado, & Chang, 2010). Despite this low completion rate, students from these historically underrepresented groups arrive at United States colleges and universities with equivalent amounts of interest and excitement in STEM as their peers, yet are significantly less likely to persist in STEM majors (Maton, Pollard, McDougall Weise, & Hrabowski, 2012), suggesting that barriers along the way may impede progress for these students.

Likewise, recent data shows that first-generation college students (FGCSs), defined as students who enrolled in postsecondary education, but whose parents had no such experience, are far less likely to graduate with a bachelor’s degree than their continuing generation peers (i.e., one or more parents with postsecondary experience; Permzadian & Credé, 2016; Redford & Mulvaney Hoyer, 2017). Indeed, nearly 90% of FGCSs in the United States do not graduate within 6 years of enrollment (Saenz, Hurtado, Barrera, Wolf, & Yeung, 1971).

Demographic variables may have some utility in explaining differential outcomes, but caution must be made when including these variables as features in predictive models. First, the use of demographic data has the potential for perpetuating prejudices (Tene & Polonetsky, 2013). Second, a student’s demographic variables have limited practical utility as they are immutable characteristics that cannot serve as targets for intervention (i.e., one’s age, racial, and cultural affiliations are fixed, personal-level characteristics). There also is the possibility that additional data that characterize students’ engagement during learning subsume demographic variables once they are added to models. An opposite outcome is also possible, where demographic variables explain a high degree of variability in the outcome measure that results in the removal of changeable behavioral variables in the prediction model. This might wash out important behaviors that occur across specific demographic categories, thus limiting utility in gaining insights from data. Third, the richness of the behavioral data should suffice. The difficulties specific populations have in academic arenas are not a direct result of their demographic background but rather are a function of their behaviors and societal biases or pressures. Taken together, the inclusion of demographic variables may interfere with the usefulness of the behavioral models.

This is not to say that demographic data should be ignored or not used at all in prediction modeling. Rather, it is vital that demographics be used in a very specific and narrow way: to ensure equitable prediction across demographic categories. The predictive model must predict individuals across racial/ethnic, gender, and generation status equally well (Bernacki et al., 2020). Teachers and researchers need to make sure that representation exists across all categories to ensure that the resulting predictions serve all students rather than unintentionally exclude certain populations from potential interventions (Slade & Tait, 2019). At present, this approach to the analysis of demographic/student background characteristics is not the norm. Most of the papers we reviewed above did not assess model accuracy for the subgroups of students on whom most research and support efforts in STEM education are focused: women, members of ethnic minority groups, and first-generation learners are those underrepresented in the STEM workforce. As two recent exceptions, Lee and Kizilcec (2020) and Yu et al. (2020) evaluated model performance in subpopulations of historically disadvantaged students including women, ethnic minorities, students from low-income families and first-generation college students. The authors found that these student populations were prone to algorithmic biases across prediction models. For example, the models overestimated women’s course success (Yu et al., 2020) and underestimated ethnic minorities’ course success (Lee & Kizilcec, 2020). Hence, the accuracy predictive models obtain hence needs to reflect those models’ ability to predict the outcome of a hypothetical student regardless of a demographic subgroup a student belongs to.

## Analytic considerations

### Machine learning algorithms

Researchers may not always know the best model to use to predict student success. It may be unknown which features are the best to include, which functional form they take when relating to the outcome, and if the prediction on one data set would work equally well for new data. For this reason, machine learning algorithms are often used to arrive at a model that provides the best prediction of the outcome given the features available. Even in the case where there are strong theoretical reasons for including a variable, such as those derived from a specific learning theory, machine learning approaches will likely still offer benefits by selecting the variables that optimally predict without overfitting the model to the data.

It is important to keep in mind the end goal of prediction—which is to predict. Variables that might not be of substantive interest might end up being very predictive of outcomes. For example, doing well on the first homework assignment might, in some cases, be predictive of the distal outcome of their final grade in the course. There is little room to intervene here, and this variable might not hold much substantive relevance to educational learning theories. Even when lacking substantive interpretation, it is important to include these types of predictors if they are ethical and if they improve the model’s prediction.

The majority of machine learning algorithms used for the purpose of prediction are considered embedded machine learning methods. This means that the learning occurs simultaneously with feature selection. Commonly used examples in psychology are decision trees (Breiman, Friedman, Stone, & Olshen, 1984; Kass, 1980) and regularization approaches such as Least Absolute Shrinkage Selection Operator (LASSO; Tibshirani, 1996) and elastic net (Zou & Hastie, 2005). We focus here on results from one common approach, elastic net, as this approach is easily interpretable and thus will aid in discussion of key decisions regarding feature selection. By using one analytic approach, we can better highlight the importance of feature manipulation and selection with a real data example. There are many options available to researchers and an exhaustive and comprehensive overview is outside the scope of the present paper. We end this section with a brief synopsis of available methods with the goal of providing researchers with a set of questions to ask themselves when choosing a prediction algorithm.

Elastic net is an estimation approach that can be used for a variety of models. Because most researchers are familiar with the generalized linear model, here we describe estimation for the simplest case – that of a linear regression. In practice, researchers may seek to predict the final grade of an individual on a continuous scale, in which case linear regression is an appropriate model. Should researchers wish to predict grade categories such as pass or fail or the letter grade, logistic or multinomial regression would be better models to use.

#### Elastic net

We describe here elastic net from within a regression framework. Assuming an intercept of zero, those familiar with linear regression will recognize that the cost function that is minimized is:

1 $$\sum\limits_{i=1}^{N}(y_{i} - \sum\limits_{j}\beta_{j}x_{ij})^{2}$$

where the term within the parentheses are the equation residuals. The goal is to minimize the squared errors. We start with LASSO as this is the foundation for elastic net. In LASSO, this cost function is subject to the penalty:

2 $$\sum\limits_{j}|\beta_{j}|\leq t$$

where *t* ≥ 0 is the tuning parameter that controls the amount of shrinkage applied to estimates. Simultaneous estimation and variable selection are conducted by minimizing:

3 $$\sum\limits_{i=1}^{N}(y_{i} - \sum\limits_{j}\beta_{j}x_{ij})^{2} + \lambda_{1}\sum\limits_{j}|\beta_{j}|$$

When *λ*_1_ = 0, the estimates are the same as least square estimates as there is no penalty. Those familiar with ridge regression may see some similarities. The difference is that LASSO uses an *l*1-norm penalty |*β*| whereas ridge regression uses the *l*2-norm penalty by squaring the term within the summation on the right *β*^2^. Unlike ridge regression, lasso regression is capable of shrinking some parameters to zero, thus making it an ideal approach for feature selection in a data-driven manner.

Elastic net includes both ridge regression and LASSO penalties by minimizing:

4 $$\sum\limits_{i=1}^{N}(y_{i} - \sum\limits_{j}\beta_{j}x_{ij})^{2} + \lambda_{1}\sum\limits_{j}|\beta_{j}| + \lambda_{2}\sum\limits_{j}{\beta_{j}^{2}}$$

A benefit of elastic net over LASSO is that, if multiple variables are correlated, LASSO tends to just choose one of them. This is opposite what occurs in the case of ridge regression, where the coefficients of correlated predictors are shrunk towards each other. For this reason, elastic net has become the preferred method for simultaneous variable selection and estimation. Simulation studies have supported that elastic net outperforms LASSO in terms of recovering the true effects that relate to the outcome (Ogutu, Schulz-Streeck, & Piepho, 2012; Waldmann, Mészáros, Gredler, Fuerst, & Sölkner, 2013; Zou & Hastie, 2005).

#### Other methods to consider

We chose elastic net as our prediction model because it not only optimizes how many and which features result in the most predictive model, but it also allows for interpretable results in the form of regression coefficients that will be helpful for pedagogical purposes here. There are, however, many other methods we could have chosen if prediction was our primary goal and understanding of the relationship between online behaviors and likelihood of success was inconsequential. Here, we briefly review popular machine learning methods for prediction and in which situations they may be useful.

Popular machine learning methods for classification problems (i.e., did a person pass or fail?), other than elastic net, are neural networks, naive bayes, k-nearest neighbors, decision trees, random forests, and support vector machines (Kotsiantis, Zaharakis, & Pintelas, 2006; Osisanwo, Akinsola, Awodele, Hinmikaiye, Olakanmi, & Akinjobi, 2017). That this is a partial list emphasizes the large number of available machine learning methods. When prediction is the goal, many researchers simply fit as many models as possible and determine which one performs best at prediction. Some models, however, may be more useful in different situations.

##### Neural networks

(McCulloch and Pitts, 1948; Rosenblatt, 1958) are a modeling technique biologically inspired by the action of neurons in the brain (Rumelhart, Hinton, & Williams, 1986). Neural networks utilize the nonlinear relationships underlying a data set to perform classification. A neural network consists of many ”neurons”, nodes, each of which is a mathematical function similar to regression that contributes to the classification decision. Neural networks typically consist of several layers of these nodes, with the output layer being the classification layer. Similar to regression, hidden layers, or layers between the input and output layers apply weights to the input. Weights are optimized in order to minimize classification error. Neural networks can be applied to both categorical and continuous predictors and outcomes and scale well to large sample sizes. Model interpretation, however, is difficult, and is often referred to as a ”black box” (Castelvecchi, 2016) because model parameters are not inherently interpretable due to overparameterization. Montavon, Samek, & Müller (2018) provide methods for interpreting neural networks.

##### Decision trees

(Breiman et al., 1984; Kass, 1980) are another popular method. A decision tree consists of a binary tree where each node has a threshold, determining which node is next traversed. The final, or terminal, node determines classification of a case. Decision trees are computationally fast for small to medium datasets, often accurate, and can be adapted as a regression tree when the outcome is continuous. In simple cases, decision trees are useful for visually understanding how different variables impact an outcome and provide useful interpretation since the top nodes in a tree are the most important variables in the classification process. In cases with many branches, or decisions, interpretability and accuracy is reduced. Finally, they are non-linear, allowing for less strong assumptions than regression techniques, and are potentially less sensitive to outliers than linear regression methods (Murthy, 1998).

##### Random forests

are an extension of decision trees that use the concept of bagging (i.e., getting the average model of several models) to improve model performance (Breiman, 2001). Here, bootstrapping is performed to create decision trees from many random samples in the training set. Prediction on a new data point is performed by predicting the class in every decision tree created, then averaging the results to determine the final result. Random forests have many of the same properties as decision trees. They have a lower sensitivity to outliers, allow for identification of the most important features, and are computationally efficient. They improve upon decision trees by reducing overfitting, having robustness to noise, and improving classification accuracy (Breiman, 2001). One drawback, however, is that they bias categorical variables that have many levels (Strobl, Boulesteix, Zeileis, & Hothorn, 2007).

##### Naive Bayes

is another simple technique that relies on calculations based on two probabilities, the probability of each class (*y*) and the conditional probability (*y* given *x*) (Lewis, 1998; Rish & et al. 2001). Naive Bayes, however, comes with the strong assumption that all *x* values are independent. That all *x* values are independent is unlikely in the context of LMS behaviors where, according to learning theory, predictors will likely be dependent on one another. If naive Bayes, however, works well as a predictive algorithm, and prediction is the primary goal of the model, researchers may choose to ignore this assumption. Because of its computational simplicity, naive Bayes works well with large training sets and many predictor variables. Additionally, naive Bayes can be used in multi-class classification problems and works especially well when the predictor variables are categorical.

##### K-nearest neighbors

(Cover and Hart, 1967) is a technique where prediction of a new case is determined by summarization of the *k* nearest cases. In *k*-nearest neighbors, the algorithm is used on the entire data set, instead of a training and test set. Typically, closeness is determined by Euclidean distance (Zhang, 2016). *K*-nearest neighbors often requires dimensionality reduction prior to running the algorithm due to the difficulty of measuring distance with high numbers of dimensions (Beyer, Goldstein, Ramakrishnan, & Shaft, 1999).

##### Support vector machines

(SVMs; Cortes & Vapnik, 1995) are a powerful modeling technique with high accuracy. SVMs work by finding the hyperplane, which can be linear or non-linear (e.g., a line in two-dimensional data), that best separates the data into classes. SVMs are optimized on the margin between the points closest to the hyperplane, where larger margins are optimal. SVMs are very efficient at classifying new data but slower to train than some of the other methods mentioned here (Cristianini, Shawe-Taylor, & et al. 2000). Additionally, this technique does not make strong assumptions, unlike regression techniques and naive Bayes, and typically, does not result in overfitting due to regularization. One potential drawback, depending on the aims of the researcher, is that the standard SVM does not shrink low values to zero. Instead, all variables are included in the model, meaning that researchers will not have a reduced list of predictive variables that could be targeted via intervention. If the aim is selection of relevant variables, regularization approaches or using regularization on the SVM are better suited.

### Evaluating model performance

Performance measures are an important choice in machine learning. Again, which metric should be used often depends on the goal of the research. For example, if misclassifying a case as a false negative carries greater risk than misclassifying a case as a false positive, one might choose to prioritize sensitivity over other measures. Relying solely on sensitivity, however, would result in a model where all individuals are classified in the positive group, which is not a practical or useful model. In this section, we review several measures of model performance and discuss the pros and cons of using each to choose the best model from a set of fitted models. Ultimately, one should run several models using several different performance measures, but choosing among the models can be aided by prioritizing certain performance measures over others.

Before discussing the performance measures, we first define the following terms: true positive (TP), false positive (FP), true negative (TN), false negative (FN). A true positive is a case classified as positive that is also positive according to ground truth. Conversely, a false positive is a case classified as positive that is negative according to ground truth. For example, a student classified as being at risk for a lower grade who actually performed well in the course would be a false positive. Similarly, true negatives are cases classified as negative that are actually negative, and false negatives are cases classified as negative that are actually positive (i.e., a student classified as not being at risk for a lower grade who performs below threshold). Counts of these values factor into all performance measures we present here, though this is not an exhaustive list.

#### Accuracy and misclassification rate

Accuracy is interpreted as the proportion of correct classifications out of total classifications (Baratloo, Hosseini, Negida, & El Ashal, 2015). More specifically:

5 $$Accuracy = \frac{TP + TN}{TP +TN +FP +FN}$$

Users should be careful about interpreting high accuracy values with class imbalanced data. In data where there are few cases of the negative class, a model which predicts all cases to be in the negative class would achieve high accuracy but have no predictive value (Longadge & Dongre, 2013).

Misclassification rate, however, is the proportion of incorrect classifications out of total classifications.

6 $$Misclassification = \frac{FP + FN}{TP +TN +FP +FN}$$

Care should also be taken in interpreting misclassification rate with class imbalanced data (Krzanowski, 2005).

#### Sensitivity and specificity

Sensitivity is defined as the proportion of positive cases correctly classified as positive. Sensitivity is sometimes also referred to as recall or the true-positive rate and is defined in equation form as:

7 $$Sensitivity = \frac{TP}{TP + FN}$$

Conversely, specificity is the proportion of negative cases correctly classified as negative. Specificity is sometimes referred to as selectivity or the true negative rate and is defined in equation form as:

8 $$Specificity = \frac{TN}{TN + FP}$$

A perfect classification model would have a sensitivity of 100% with a specificity of 100%. Ideally, the chosen model would balance these two statistics. It would be easy to create a model with either 100% sensitivity or 100% specificity by classifying all cases as either positive or negative. However, this type of model would have no predictive value (Baratloo et al., 2015). In our work, we do not choose the model based on sensitivity or specificity because of this issue. We also do not select models based on misclassification rate because of the class imbalance issues. We do, however, use accuracy as a model selection measure in order to have one type of performance measure from this class of performance measures.

#### Cohen’s kappa

Kappa (Cohen, 1960) is a useful measure for both multiclass solutions and imbalanced classes, where accuracy can be misleading (i.e., cases where one class is underrepresented in the sample). Kappa can be interpreted as a measure of how well the classifier is performing compared to a model that just classifies cases at random (Landis & Koch, 1977). Originally developed as a measure of interrater reliability, the formula for kappa is:

9 $$\kappa = 1 - \frac{1-p_{o}}{1-p_{e}}$$

where *p*_*o*_ is the accuracy (i.e., agreement between ground truth and classifier) and *p*_*e*_ is the expected agreement (i.e., accuracy if model classifies at random). The highest possible value of kappa is 1, where 1 represents perfect classification. Cutoffs have been provided by Landis and Koch (1977), where 0–0.20 is slight agreement, 0.21–0.40 is fair agreement, 0.41–0.60 is moderate agreement, 0.61–0.80 is substantial agreement, and 0.81–1 is almost perfect agreement. A negative value would indicate that the classification model is worse than random.

#### AUC-ROC

The area under the receiver-operator characteristic curve (AUC-ROC; Melo, 2013) is a measure that balances the previously defined false- and true-positive rates. The ROC is a curve where the false-positive rate is plotted on the *x*-axis, and the true-positive rate is plotted on the *y*-axis. By exploring the ROC, one can determine how an increase in false-positives determines an increase in true-positives, the goal being to determine the probability of true positives at different thresholds of false positives. For example, we could have a true-positive rate of 100%, but in doing so, we would also have a false-positive rate of 100%. The AUC is the area under the ROC and ranges from 0.5 to 1. The closer the AUC is to 1, the better the model is at reducing both false-positives and false-negatives.

#### Algorithmic fairness

In addition to using these measures of model performance to assess overall model performance, we also suggest using these measures to explore model performance among subgroups within one’s dataset to determine if the model is performing equitably among these subgroups. Kizilcec et al. (2021) described three definitions of algorithmic fairness across groups: independence, separation, and sufficiency. Independence means that algorithmic decisions should be independent of group status. To meet independence, the proportion of individuals predicted to be in the positive class should be equal across groups. Separation means that the algorithmic decisions should be independent of group status, conditional on true predictions. That is, true-positive (sensitivity) and false-positive rates should be equal across groups. Under sufficiency, the proportion predicted to be in the positive class for a given group should equal the proportion that is actually in the positive class. Kizilcec and Lee (2021) described sufficiency as a ”weak” guarantee of algorithmic fairness.

### Oversampling

Oversampling is used when one of the outcome categories is much larger than the other, such as when researchers would like to increase the weight of the smaller population. In data where one of the predictive categories is rare, it is possible that machine learning approaches return models where all individuals are assigned to one class, resulting in either high specificity with poor sensitivity or poor specificity with high sensitivity. If the sample is balanced, it is likely that oversampling is not necessary. As with the above analytic overviews, we do not intend this review to be exhaustive - rather, we sought to provide researchers with enough understanding to know how to consider emerging approaches. When using any oversampling method, oversampling should only be completed on the training set and not the entire data set. If oversampling is completed on the entire set, synthetic data points that are already in the training set may also be created in the test set, potentially leading to overfitting (Kovács, 2019).

Random oversampling is a ’naive’ oversampling method in which random cases from the minority class are duplicated in the training set (Ling & Li, 1998). Because the data is being duplicated in the training set, it is possible this technique leads to overfitting of the minority class (i.e., model performance is better in the training set but worse in the test set because the model is being fit to data with a larger *n* in the smaller class than actually exists). When using this method, we recommend comparing the performance of the model obtained from oversampled data to the original data in both the training and the test set to determine if overfitting is an issue (Japkowicz & et al. 2000).

Synthetic Minority Oversampling TEchnique (SMOTe; Chawla, Bowyer, Hall, & Kegelmeyer, 2002) is one of the most commonly used methods for oversampling data where at least one of the predictor or outcome categories is underrepresented. With SMOTe, overfitting is still possible but less likely than with random oversampling because SMOTe is not simply duplicating existing data. SMOTe creates new, synthetic data points using information from existing data points in the underrepresented categories. Conceptually, SMOTe creates new, synthetic data points between existing real data points in the minority class. This is done by creating a vector between *k*-nearest neighbors of each minority data point and generating synthetic data points on these vectors. There are two tuning parameters for SMOTe, *k* and *n*. *K* is the number of neighbors to connect to each data point, and *n* is the number of times to duplicate that data point. In R (Torgo & Torgo, 2013), the default for *k* is five nearest neighbors. Additionally, the minority class is oversampled at a rate of 200% and the majority class is undersampled at a rate of 200%.

## Empirical example

Here, we use an empirical example to illustrate the ideas and recommendations presented above. After selecting a model, oversampling, and evaluation methods, we can proceed with a feature selection process that is guided by learning so that our model can be both predictive of learning outcomes and potentially informative for a future intervention effort. For our empirical example, data were collected from students across multiple sections and semesters of an introductory biology course. All course sections utilized the same syllabus and LMS course site designs. Course instructors employed flipped classroom (i.e., students address topic content and assignments before the related class session) and active-learning (i.e., in-class individual and group activities and formative assessments) pedagogies delivered via a blended learning format (i.e., online resources and assignments coupled with in-person instruction). For each of 24 topic lessons, students were tasked with completing guided reading assignments and an online homework module before the related class session where formative assessment questions are interwoven into lecture presentations. Additionally, a prior knowledge assessment on the first day of class, seven multi-topic quizzes, three unit midterm exams, and a cumulative final exam were administered. Our behavioral data came from multiple sources: (1) LMS interactions; (2) use of the textbook publisher’s online assignment platform; (3) use of the course section’s forum site; (4) attendance logs for instructor office hours; (5) use of peer mentoring, and (6) use of learning center services. Any time a student took an action such as submitting assignments, downloading documents, clicking links to external websites, creating or replying to forum posts, or scheduling office hours, a timestamped log entry was created. Static data consisted of institutional data about the learner including demographic information and prior performance (i.e., not used in the model) and gradebook data about their performance in the course (i.e., used to produce the criterion variable.)

In its raw form, the data represent individual actions to access a single piece of content, respond to a single interactive exercise, or to subscribe to a single service. These events may afford predictive power in isolation, but they are voluminous to consider, and the specific combination of these very fine-grained events that are predictive for one sample may be less apt to retain their predictive accuracy when applied to future samples. Thus, we rely upon our understanding of the learning context and the instructional design intentions that motivated the provision of these resources to guide our feature engineering. That is, we aggregate events involving like objects into classes of resource type use, and where possible, tag these with the learning processes those objects were designed to afford. For this example, we utilize data collected during one semester of an introductory level course to provide concrete illustration of the types of features one might have access to from LMS trace data. We also leverage this data to demonstrate the differential predictive ability that behaviors can have based on how researchers choose to quantify and code them.

### Feature generation

As discussed in our earlier section on predictor variables, an important aspect of feature generation from trace data is deciding how to create predictive and theoretically meaningful features from raw user log data. We use this example to illustrate the many decisions involved. Specifically, we discuss the window of prediction, the use of counts versus dichotomous features, aggregation across time, and the specificity of course unit metadata. We do not go into detail regarding the data themselves as these are highly specific to the course and instructors. Thus, they may have limited generalizability. The process we use here, however, is generalizable.

#### Window of prediction

Like most decisions we discuss here, deciding which actions to extract from user logs depends on the research question. In many studies where researchers aimed to develop prediction models, researchers used the first 6 weeks of data, but there was also substantial variance in that range, with some studies limiting data collection down to the first week, whereas others continued data collection throughout a 16-week semester. If the goal is to implement a mid-semester intervention aimed at identifying students who are less likely to succeed, modeling the whole semester of data would not be useful. A better decision would be to model the early weeks of the course to provide a sufficiently timely prediction and to preserve an opportunity to intervene before an assessment that is critical to succeeding in a course. In the current study, we sought to build a model that produced an accurate prediction with time to intervene before the very first assessment in the course and explored using either the first 3 weeks or the first 4 weeks of the semester in order to determine if fewer weeks of data retained similar predictive value to more weeks of data.

#### Counts versus dichotomous variables

Next, we considered how to code information from our given window of prediction. In the current study, we coded variables as counts or dichotomous depending on how we expected a predictor to relate to the outcome. Specifically, we considered the type of event that was observed and whether that event would confer ongoing benefits, or whether a single instance of the event might be sufficient to confer benefit, with additional instances providing no clear advantage to the learner. A common distinction we made was between access of downloadable content where a single access would suffice to afford enduring use of a file versus visits to an interactive object, such as a practice quiz that afforded repeated self-testing or a forum that afforded ongoing engagement with peers. For this reason, we ran models containing solely count values and models containing a mixture of count and dichotomized variables. First, we categorized event types as best represented as dichotomous when a single instance made sense and repeated instance did not, and as continuous when ongoing use afforded ongoing benefit. Then, we examined a thresholding approach where use or disuse of a class of resources might serve as a categorical descriptor of an individual’s approach to learning in the course. These dichotomous features described a learner as one who did or did not engage in a variety of events, and at times provided additional predictive power to models when provided as candidate features. Some examples of categorical variables that explained variance over and above continuous versions included: submitting a forum question, attending a review session, attending tutoring, meeting with a writing coach, downloading class 0 lecture notes, submitting guided reading questionnaire at different points in the semester (GRQ; required homework item) 4, downloading GRQ 7, submitting a revised GRQ 1, submitting a revised GRQ 4, saving a GRQ 1, downloading lecture 0 slides, and downloading lecture 3 slides. These categorical variables describe a distinct tendency to engage in a particular behavior, and categorical tendency to engage vs. not engage in a behavior can be a more important piece of information that a representation that assumes a linear effect where each additional engagement in a behavior confers increasing benefit.

#### Aggregation across time

Similar to our method of aggregating use of similar objects into counts of access of resources that reflect a theoretically interpretable learning behavior, we also aggregated events into contextually meaningful periods of time in which they occurred. That is, university courses are often organized into content units, and those units are further broken down into activities that conform to a weekly rhythm where students engage with a prescribed set of content in each semester week. Thus, we considered whether individuals’ summative effort through the prediction window would predict their performance and whether disaggregating such effort into semester weeks might further decompose variance in the timing of these events in ways that provide additional predictive power. If we are looking at the first 4 weeks of data, do we want to get a count of each action for each week or a count that aggregates across the entire 3-week time period? If there is reason to believe that week 1 behavior would be differentially predictive than week 3 behavior, it would make sense to aggregate on a weekly basis rather than across all 3 weeks. We might hypothesize that a download of the syllabus in the first week of a course might be positively associated with strong performance in a course, whereas the same download conducted in the third week of the course might be minimally or even negatively associated with performance. If, however, parsimony is important and breaking the data down by week is not expected to add much value, simply aggregating across the entire 3-week period may be preferred. We explored both methods of aggregation along with the combination of both methods of aggregation to determine which led to better prediction in the current data set.

#### Specificity of features

As mentioned in the section on predictor variables, behaviors can be coded at varying levels of specificity. A researcher can choose to create features with behaviors as broad as a download of any resource to as specific as a download of Unit 3 lecture materials (in our course, topics were separated into units). We explored the use of features with three levels of specificity: (1) unit-specific; (2) common events; and (3) theory-driven behaviors (i.e., a further aggregation of common events that are consistently reflective of a learning strategy).

Unit-specific events tie the action to the content being learned. Thus, there can be a downloading lecture slides feature for each unit in the course, forum contribution for each unit in the course, and so on. A common event is an event that summarizes the use of a particular learning object type (e.g., access a course outline), and ignores the specific topic to which it pertains (e.g., chapter 3). Other examples of common events are downloading lecture slides, contributing to a forum, or completing a quiz. Finally, researchers can look to theory to guide how trace data can be transformed into useful features. Within the scholarship on STEM learning, researchers have often turned to self-regulated learning (SRL; Greene, Deekens, Copeland, & Yu, 2018; Hadwin, Järvelä, & Miller, 2011) theory to understand and intervene upon student behaviors. The primary tenets of SRL theory (e.g., course success requires students’ active and thoughtful pursuit of learning goals and such pursuit requires self-knowledge and the ability to monitor learning and adjust as needed; Pintrich, 2000) can be used to create predictive features. For example, syllabus access can indicate student identification of learning goals, students’ views of and responses to quiz feedback can indicate monitoring, and particular contingencies among behaviors (e.g., monitoring learning by accessing feedback and then adjusting learning by rereading sections of a course text) can be used to infer the kinds of adaptations that indicate effective self-regulation and higher likelihood of student success (Bernacki, 2018; Binbasaran Tuysuzoglu & Greene, 2015).

### Analysis

Each feature set was split into a training set (75% of observations = 265 students) and a test set (25% of observations = 88 students). Logistic regression with elastic net penalty was then applied to each data set to predict outcomes on Exam 4, a summative final exam that serves as a criterion variable representing mastery of topics covered throughout the semester. Each outcome was binarized using a ”C+” threshold. A score greater than or equal to 80 was above a ”C+” (per syllabus) and coded 0 here, while scores lower than 80 were considered ”C+ and below” and coded 1. This grade was chosen as it serves as a pragmatic discriminator where those who tend to earn grades in the A to B range move forward in their program of study, whereas those who earn grades in the C range or worse often repeat the course, either as a requirement of their degree program, or because future employers or degree programs require an A or a B for acceptance into jobs or programs.

All predictors in the test and train sets were standardized separately by mean-centering and dividing by the standard deviation. Tenfold cross-validation was used to identify the best regularization/model hyperparameters. Three different sampling techniques were used: no oversampling, random oversampling, and SMOTe. Additionally, each analysis was conducted choosing the best model on either accuracy or Cohen’s kappa. In total, 132 models were run. An ANOVA with all main effects and relevant two-way interactions was run to compare the kappa value of each model. We chose to look at kappa values to assess model performance regardless of the performance measure used in model selection during the model building stage. In addition to obtaining the accuracy, kappa, and sensitivity for each model, we also report performance measures across first-generation status, minority status, gender, honors student status, student’s major, and course section to determine the level of algorithmic fairness across groups. We also report additional statistics on independence (proportion of each group predicted to be in positive class) and separation (false-positive rates) as suggested by Kizilcec et al. (2021). Important to note is that the models with dichotomous variables only included unit-specific features. Including common features would result in the same results.

### Results

#### Comparison of models

An ANOVA with all main effects and a two-way interaction between specificity of feature and method of aggregation was completed to uncover differences in kappa depending on model performance measure, oversampling method, window of prediction, specificity of feature, whether some variables were dichotomized, and aggregation method. The single two-way interaction was included based on a preliminary model of only main effects. Results from the ANOVA are reported in Table 2.

**Table 2:** ANOVA comparing kappa values of models

	Df	Sum Sq	Mean Sq	F-value	Pr(>F)
Model Perf. Eval.	1	0.00	0.00	0.44	0.508
Oversampling Method	2	0.01	0.01	2.42	0.093
Window of Prediction	1	0.01	0.01	4.12	0.045*
Specificity of Feature	2	0.05	0.03	9.41	< 0.001***
Aggregation Method	2	0.31	0.16	58.58	< 0.001***
Dichotomized	1	0.00	0.00	1.58	0.212
Specificity of Feature:Aggregation Method	3	0.04	0.01	5.24	0.002**
Residuals	119	0.32	0.00		

Results indicate that the window of prediction, the specificity of the feature, the aggregation method, and the interaction of feature specificity and aggregation method were significantly associated with model kappa. Signif. codes: 0^′^∗∗∗^′^0.001^′^∗∗^′^0.01^′^∗^′^0.05.

Results indicate statistically significant relations between the kappa value of the models run and the main effects of window of prediction (*p* < .05), specificity of feature (*p* < .01), and method of aggregation (*p* < .001). Additionally, there was a statistically significant interaction effect between specificity of feature and aggregation method (*p* < .01). Type of performance measure (*p* = .51) and whether or not some variables were dichotomized (*p* = .21) were not statistically significant predictors.

**Fig. 1:**
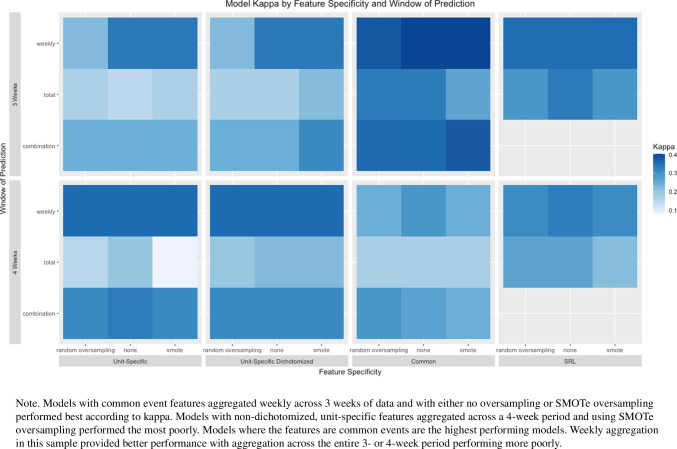
Differences in model kappa according to different model specifications

A heatmap in Fig. 1 shows differences in Cohen’s kappa depending on model specifications. Models with common event features aggregated weekly across 3 weeks of data and with either no oversampling or SMOTe oversampling performed best according to kappa. Models with non-dichotomized, unit-specific features aggregated across a 4-week period and using SMOTe oversampling performed the most poorly. Overall, the models where the features were common events appeared to be the highest performing. Additionally, weekly aggregation in this sample provided better performance with aggregation across the entire 3 or 4-week period performing more poorly, indicating that a more granular count leads to better model performance.

#### Winning model

Here, we present results from the winning model. Those interested in viewing full results from the full set of models tested can look in the supplemental materials at https://osf.io/mj4ah/. The model we present here worked best in all scenarios and for our objectives of having a predictable and interpretable model. This winning model used data from the first 4 weeks of the semester. No oversampling was completed, and the performance measure was accuracy. Unit-specific feature types, aggregated across both the entire 4-week period and by each week, were used in the model. No counts were dichotomized, and there were a total of 139 potential features.

##### Model prediction performance

The overall best-performing model achieved an accuracy of 0.75, a Cohen’s kappa of 0.49, a sensitivity of 0.74 , and an AUC-ROC of 0.75. In other words, the model was successful in classifying 75% of students into classes based on prediction of their earning of a C+ or worse or a B- or better on a final exam. The model performed 49% better than a model that would have classified all students as C+ or worse earners (i.e., assigning this label to all cases based on it being the more populous class). Most importantly, for the purposes of prediction, a sensitivity of 0.74 means that the model successfully identified 3 out of 4 students who would ultimately earn a C+ or worse on a final exam, using only those data gathered prior to the first unit exam. The model thus provides an accurate prediction at a timely moment when these students could be provided with learning support that could help them avoid having to repeat the course due to earning a C+ or worse on an early exam that contributes substantially to their final grade and which can delay or derail STEM degree attainment and workforce entry. Group distributions and model performance metrics for each group can be found in Table 3.

**Table 3:** Model performance broken down by demographic groups and course sections

	Accuracy	Kappa	Sensitivity	FP Rate	Predicted Positive
First generation (*n* = 17)	0.65	0.16	0.69	67%	76%
Other generation (*n* = 62)	0.76	0.52	0.74	20%	44%
Underrepresented minority (*n* = 21)	0.71	0.30	0.80	50%	71%
Non-minority (*n* = 55)	0.75	0.48	0.70	22%	42%
Female (*n* = 56)	0.70	0.39	0.70	32%	52%
Other (*n* = 33)	0.83	0.66	0.82	15%	33%
Honors student (*n* = 6)	1.00	1.00	1.00	0%	0%
Non-honors student (*n* = 74)	0.72	0.43	0.70	29%	50%
Biology major (*n* = 18)	0.78	0.53	0.82	29%	61%
Non-biology major (*n* = 62)	0.73	0.45	0.69	26%	47%
Section 1 (*n* = 51)	0.73	0.42	0.65	23%	39%
Section 2 (*n* = 36)	0.78	0.53	0.82	29%	61%

Model performance was similar across course sections and across biology majors and non-biology majors, with slightly better performance in section 2 and in biology majors. The model performed perfectly among honors students. There is slightly better performance in non-minorities. There is also better performance in non-female students. Finally, there is moderate performance of the model for first generation students, indicating a deficit in the model to classify first generation students.

##### Model equity performance

There was variability in model accuracy and kappa across subgroups. That said, except for first-generation students, we attained accuracy greater than or equal to 0.70 and Kappa greater than or equal to 0.30 across all groups. In general, the model performed similarly across course sections and across biology majors and non-biology majors, with slightly better performance in section 2 and for biology majors according to accuracy and kappa values. The model performed perfectly among honors students (i.e., all students were correctly classified). However, there were only six honors students, and non-honors classification performed well. Concerning demographic groups, we observed slightly better model performance based on accuracy and kappa in groups traditionally overrepresented in STEM. There was also better model performance in non-female students. Overall model performance for members of underrepresented racial minority groups (*accuracy* = 0.71, *κ* = 0.30) and among female students (*accuracy* = 0.70, *κ* = 0.39), however, was good. In constract, the model achieved only moderate performance for first generation students, indicating a deficit in the model (*accuracy* = 0.65, *κ* = 0.16) .

Based on the sensitivity, false-positive rates, and percent of individuals predicted to be in the positive class, there was variability in our model regarding the requirements of independence and separation. The greatest discrepancies (excluding the honors student comparisons) were between first-generation and continuing-generation students and between underrepresented racial minority groups and those in the majority group. The deficit in model performance combined with the lack of independence and separation indicate a need to investigate different ways of predicting the success of first-generation students as well as underrepresented minorities.

#### Important features

**Fig. 2:**
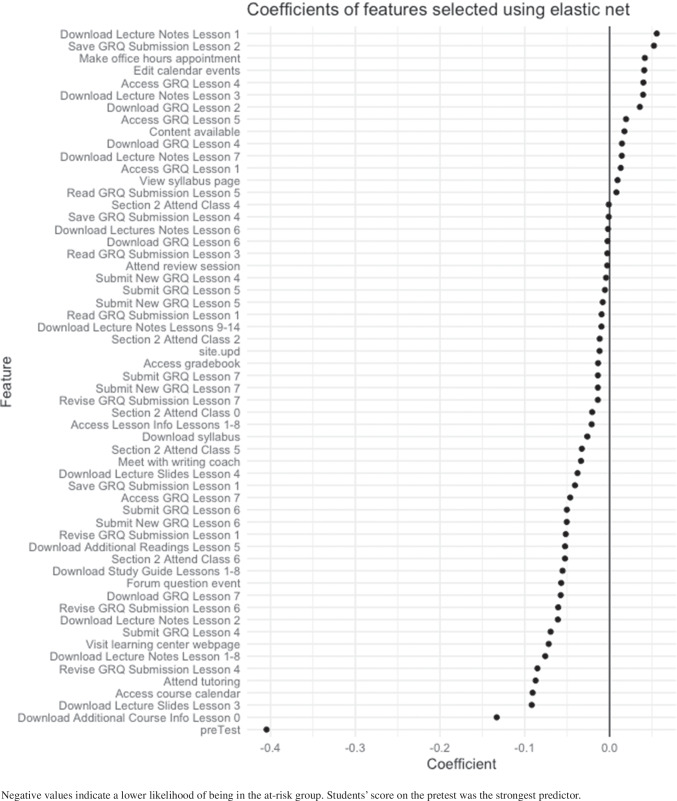
Coefficient strength and direction for winning model

In the winning model, 58 features were selected for inclusion in the model. A summary of presence of feature and strength of relationship with exam 4 outcome are presented in Fig. 2. An inspection of the features reveals the kinds of approaches to learning that were associated with successful or poorer performance, and these insights can inform future methods to support learners. For the majority of features selected, higher counts of engagement with a given action resulted in a lower likelihood (i.e., negative coefficient) of the student being classified as performing poorly in the class. Examples of the most important types of engagement (i.e., those with high absolute coefficient values) for this increased likelihood of acceptable academic performance in the course were increased counts of: downloading additional course information prior to the start of the course, downloading the lecture slides for lesson three, accessing the course calendar, and attending tutoring. Performance on the pretest had the greatest impact, indicating that prior knowledge is important to course outcome. For a few variables, greater engagement was associated with a higher likelihood of poor performance in the course. Examples of engagement associated with increased risk were increased counts of: downloading lecture notes for lesson 1, saving GRQ submission for lesson 2, making an office hours appointment, and editing calendar events.

## Discussion

Our main goal was to create a guide for behavioral researchers interested in using digital trace data. We did this by providing a primer on methods for analysis and feature generation. We also set out to examine how different prediction modeling decisions would impact the accuracy obtained by modeling students’ early behaviors as predictors of success on a cumulative final exam in a high enrollment, gateway, science course. Overall, model performance was improved when we aggregated features on a weekly basis rather than across the entire 3- or 4-week period. This indicates that the granularity and timing of when a student completes an action is important to predicting their success. This finding follows logic, as completing assignments early or downloading the syllabus early in the semester may be associated with proactive behavior in general, thus leading to greater likelihood of success.

A third key aim of the paper was to not only accurately predict student achievement using an optimal method of modeling using learning behaviors, but also to reserve the demographic data often used to predict achievement instead as a lens to appraise the equity of model accuracy. This method allowed us to avoid perpetuating biases induced by use of person-level characteristics as indicative of future success, ensured that all variance explained by a model would owe to behaviors that could be conducted by any student and observed by researchers, and potentially inform efforts to support those predicted to perform poorly. Our best model achieved accuracy above that typically achieved by prior researchers including those who relied upon demographic variables as predictors, and this accuracy was retained for all groups to whom the model was applied during testing. This confirms that the model could be applied with the confidence that it not only avoids perpetuating biases, but also affords an equitably accurate projection of a students’ performance based only on their learning behavior for both those adequately- and under-represented in science-learning contexts and fields.

The fourth aim of the paper was to confirm that the predictive accuracy was sufficient for the subclass of students who performed poorly on the exam that served as our criterion variable. Our most accurate model was able to identify three out of four students who would ultimately go on to earn a C+ or worse on their cumulative final exam - solely based on behaviors in the course obtained prior to the first exam. In doing so, the model demonstrated a sufficient sensitivity to detect such students at a time when they had yet to begin achieving poor outcomes on assessments, and the behavior-based predictions provided some transparency into the kinds of learning behaviors that might contribute to poor outcomes and could be addressed by intervention.

### Non-malleable factors

In the field of learning analytics, the decision whether to include person-level demographic variables and other characteristics of the individual that are not amenable to intervention is the focus of ongoing discussion (Buckingham Shum, 2020) Those who advocate for their inclusion tout the contribution to the accuracy of prediction, and the explanation of variance in outcome variables based on known sociological factors. However, others argue that including these variables has the potential to overassign likelihood of a particular outcome to individuals based on factors that are entirely unrelated to the ways that a particular individual engages in learning. The inclusion of such variables has the potential to subsume variance that could be more coherently explained if it remained available to the collection of behavioral variables that might combine to explain similar amounts of variance and produce a far more interpretable model (Bernacki et al., 2020). This latter approach, while it may produce slightly less accurate prediction, may provide sufficient model performance to afford intervention, and further offer to intervene to support learners, based on the types of behaviors that they may (fail to) conduct and which are predictive of poorer outcomes.

### Implications

There are several implications to be taken from this work. First, there are many combinations of analytic and feature generation methods that a researcher could take when analyzing digital trace data. Selection of methods is going to be context specific, and the goals of the research should be well thought out. Throughout the paper, we discussed:

- the trade-off between prediction and interpretation
- the presence of class imbalance and how that should be addressed when selecting model performance techniques and oversampling methods
- choice of model considering computational efficiency, sample size, types of predictors and outcomes, and interpretability
- the decision of whether to include demographic variables
- the granularity at which features are generated
- assessing equity across groups

Our results suggest that regression with the elastic net penalty provides a model that achieves predictive power, while also allowing for simple interpretation in the regression framework. Because we achieved the best model performance with counts aggregated weekly, it may suggest that researchers could benefit from creating more granular features. With regards to other modeling hyperparameters we discussed, we suggest researchers try them all when creating predictive models to determine which ones provide the best model performance. Decisions about quantitative methods should also be made with an ethical lens (Panter & Sterba, 2011). Also, we suggest that researchers evaluate model performance based on how the model performs across demographic subgroups to ensure model equity.

### Limitations

All prediction models are necessarily context specific. They are defined by the target outcome adopted as a criterion variable. The model that is produced is predicated on the candidate features provided by the learning environment and the selection of a sample of learners who engage within it. In that sense, this study is thus limited to a single course at a single institution, and the sample that could be drawn from a pair of sections of a Biology 101 course. With that in mind, the course is a reasonable exemplar of the high enrollment gateway courses in institutes of higher education. Such work is context specific, should be replicated in additional contexts, and future researchers should experiment with elements of the design with an aim to improve upon them.

Our models generally delivered equitable accuracy to nearly all student groups, but one group whose performance the model struggled to accurately classify was first generation college students. It may be the case that these students engaged with the learning resources in ways that differed from those who may have benefited from the social influence of others who had attended university. Additional research will need to be conducted to derive larger samples and examine differences in the behaviors and potentially the intentions across first- and continuing-generation students. It is possible that first-generation students use different strategies for success or that certain strategies work better for them because of differing prior knowledge. To ensure that we provide equitable assistance to students who are likely to perform poorly academically, we need to better understand these differences.

In this study, we did not consider modeling techniques that may have better predictive value but offer little in terms of interpretation. Our priority was to obtain predictive accuracy but to also ensure that the model derived accuracy from interpretable features that could provide insight to the design of future intervention. If we only cared about prediction, we would want to include other models in the selection process (e.g., NNs, SVMs, etc.). These models have the capacity to outperform the models reported in this paper, and could be useful in future prediction efforts, especially if intervention methods have already been prepared.

### Conclusions and future directions

In this paper, we aimed to probe the optimal design choices to produce accurate and equitable prediction of student success that afforded opportunity for and insight into intervention. We achieved model accuracy superior to typical levels reported in the literature and further confirmed that this level of accuracy was achieved for both well- and under-represented groups in STEM, who are often at a disadvantage in STEM learning contexts and who are often further disadvantaged by AI decisions that perpetuate biases. We avoided such perpetuation by developing a model without person-level data describing students’ demographic characteristics, demonstrating that it is possible to model student success agnostic to a student’s background, and that focusing on their behavior can provide equitable opportunities for supporting learnings and insight into how best to do so.

In addition, we explored a wide range of feature mapping possibilities. Future research could expand upon the current paper by considering alternative methods of feature generation. Here, we represented features as counts of actions in a given unit, aggregated across the predictive window. There are endless other options to explore. Of particular interest is the use of factor analysis and other profiling methods.

Factor analysis, among other clustering and profiling methods, would provide an avenue to reduce dimensionality and to reduce error in measurement. It may also be a way to model relationships more aligned with learning theory and create more intuitive interpretations of the model. For example, knowing that downloading the syllabus early in the semester is associated with higher likelihood of success is only useful if we can understand the underlying characteristics that lead to such measurable behaviors. Combining indicator variables to measure proactiveness, however, would be more informative for developing generalizable interventions not tied to the specifics of the course modeled in the current study.

Because of the sensitive nature of the data, none of the raw data is available. We provide the code we used along with synthetic data for illustrative purposes at https://osf.io/mj4ah/. We also have the results and code for the model comparisons reported in the paper. None of the models were preregistered.
